# The chlamydial functional homolog of KsgA confers kasugamycin sensitivity to *Chlamydia trachomatis *and impacts bacterial fitness

**DOI:** 10.1186/1471-2180-9-279

**Published:** 2009-12-31

**Authors:** Rachel Binet, Anthony T Maurelli

**Affiliations:** 1 Department of Microbiology and Immunology, F. Edward Hébert School of Medicine, Uniformed Services University of the Health Sciences, 4301 Jones Bridge Road, Bethesda, MD 20814-4799, USA; 2 Current address: U.S. Food and Drug Administration, Office of Regulatory Science, 5100 Paint Branch Pkwy, College Park, MD 20740, USA

## Abstract

**Background:**

rRNA adenine dimethyltransferases, represented by the *Escherichia coli *KsgA protein, are highly conserved phylogenetically and are generally not essential for growth. They are responsible for the post-transcriptional transfer of two methyl groups to two universally conserved adenosines located near the 3'end of the small subunit rRNA and participate in ribosome maturation. All sequenced genomes of *Chlamydia *reveal a *ksgA *homolog in each species, including *C. trachomatis*. Yet absence of a S-adenosyl-methionine synthetase in *Chlamydia*, the conserved enzyme involved in the synthesis of the methyl donor S-adenosyl-L-methionine, raises a doubt concerning the activity of the KsgA homolog in these organisms.

**Results:**

Lack of the dimethylated adenosines following *ksgA *inactivation confers resistance to kasugamycin (KSM) in *E. coli*. Expression of the *C. trachomatis *L2 KsgA ortholog restored KSM sensitivity to the *E. coli ksgA *mutant, suggesting that the chlamydial KsgA homolog has specific rRNA dimethylase activity. *C. trachomatis *growth was sensitive to KSM and we were able to isolate a KSM resistant mutant of *C. trachomatis *containing a frameshift mutation in *ksgA*, which led to the formation of a shorter protein with no activity. Growth of the *C. trachomatis ksgA *mutant was negatively affected in cell culture highlighting the importance of the methylase in the development of these obligate intracellular and as yet genetically intractable pathogens.

**Conclusion:**

The presence of a functional rRNA dimethylase enzyme belonging to the KsgA family in *Chlamydia *presents an excellent chemotherapeutic target with real potential. It also confirms the existence of S-adenosyl-methionine - dependent methylation reactions in *Chlamydia *raising the question of how these organisms acquire this cofactor.

## Background

Ribosomes are complex macromolecular machines that are found in abundance in all cells that are actively making proteins. Two-thirds of the ribosome is composed of RNA molecules (rRNA) that share a high degree of conservation in primary sequence as well as in secondary and tertiary structural elements across kingdoms [1]. Numerous nucleotide modifications, mainly but not exclusively via methylation, are found on rRNAs but the functional importance of these post-transcriptional modifications remains unclear [2]. For example, the two adenosine residues in the loop of helix 45 near the 3'end of the small subunit rRNA (positions 1518 and 1519 of the 16S rRNA in the *Escherichia coli *numbering system) are universally conserved, and are dimethylated by a specific enzyme belonging to the rRNA adenine dimethylase family, which is represented by the *E. coli *KsgA protein [3]. While these enzymes are present in all three kingdoms of life including mitochondria and chloroplasts [4], a few KsgA orthologs adopted additional roles within the cell, serving for example as a transcription factor in mitochondria [5] or an essential ribosome biogenesis factor in yeast [6]. However, dimethylation is generally not essential for growth at optimal temperature.

The order *Chlamydiales *forms a deep lineage of obligate intracellular bacteria, infecting free-living amoebae, various invertebrates and all the vertebrates, and includes four families: *Chlamydiaceae*, *Parachlamydiaceae*, *Waddliaceae*, and *Simkaniaceae *[7]. The *Chlamydiaceae *are well known agents of multiple diseases in animals and in humans, with two species, *C. trachomatis *and *C. pneumoniae*, being pathogenic for humans. *C. trachomatis *is recognized as the most prevalent cause of bacterial sexually transmitted infections worldwide and, in underdeveloped nations, is also responsible for trachoma a potentially blinding disease. *C. pneumoniae *is a significant agent of respiratory disease in adolescents and adults and also is associated with cardiovascular diseases. The other chlamydial species are less relevant to human medicine, although severe zoonotic diseases in humans are caused by *C. psittaci*, *C. abortus *and *C. felis*. Species of *Parachlamydiaceae*, *Waddliaceae*, and *Simkaniaceae *are also suspected to be involved in human infections [8].

In addition to being obligate intracellular Gram-negative bacteria, all chlamydiales share a unique biphasic developmental cycle. The environmentally stable, metabolically inert and infectious elementary bodies (EBs) enter susceptible host cells and convert into the replicating and metabolically active but noninfectious reticulate bodies (RBs) inside cytoplasmic vacuoles (also called inclusions) before converting back into EBs from 18 to 48 hours post-infection depending on the species, and exiting the host cell to repeat the cycle [9]. While effective antibiotic therapies are available to treat chlamydial infections, it is becoming clear that the low number of rRNA operons in these bacteria presents an actual risk for emergence of resistance against the current preferred therapies of tetracycline or azithromycin [10,11]. Then, similar to other bacteria, targets for the development of new antimicrobials need to be identified.

The evolution of *Chlamydia *as an obligate intracellular pathogen has been associated with loss of genes encoding functions that became redundant within the host during the adaptation to parasitic/symbiotic lifestyles, similarly to mycoplasmas, phytoplasmas, α- and γ-proteobacteria [12]. Sequencing data available for eleven *Chlamydia *species reveal the presence of a KsgA dimethyltransferase homolog, yet they all lack the S-adenosyl-methionine synthetase that is required for synthesis of S-adenosyl-methionine, the donor of methyl groups in all methylation reactions. This raises the question of whether the *ksgA *homolog encodes a functional methylase in these organisms. The lack of tools for genetically manipulating *Chlamydia *has been a major barrier to the analysis of their putative virulence genes [13] and characterization of chlamydial genes has mainly relied on expression in a heterologous host system such as *E. coli*. In this study, we show that the chlamydial KsgA protein is able to functionally replace the orthologous enzyme in *E. coli*, indicating that they share the same activity, i.e. specific methylation of the small subunit ribosomal RNA. KsgA activity conferred sensitivity to the antibiotic kasugamycin (KSM) in *Chlamydia*, similarily to *E. coli*, and we were able to isolate low level KSM^R ^mutants in *C. trachomatis *which contain a frameshift mutation in *ksgA*. These mutant bacteria were severely impaired for growth, highlighting the critical role KsgA plays in *Chlamydia *biology.

## Results and discussion

### 1-Characterization of the chlamydial KsgA orthologs and their phylogenetic relationship with other family members

A gene encoding a protein highly similar to the dimethyltransferase KsgA is present in the genomes of all sequenced *Chlamydia*. Using degenerate primers, we were amplified a 5 kb-genomic region of *C. psittaci *6BC carrying the *ksgA*-like gene. *ksgA *is the second gene of a predicted bicistronic operon in *Chlamydia *[14], downstream of a gene, named *ct355 *in *C. trachomatis *serovar D, with no homology outside the chlamydiae lineage. CT355 ORF shows 59 to 100% homology amongst the *Chlamydiacee *and 26% with the protochlamydial pc0396 ORF which precedes the protochlamydial *ksgA *gene.

Genomic transcriptional profiling of *C. trachomatis *serovar D showed that both *ksgA *and *ct355 *genes are transcribed by 8 hours post-infection when the bacteria are in the metabolically active stage of development [15]. Surprisingly, while most *ksgA *orthologs use the AUG canonical translation initiation codon, an alternative *ksgA *start codon is predicted amongst the *Chlamydiaceae*, either GUG in *C. trachomatis, C. muridarum *and *C. pneumoniae *or UUG in *C. psittaci *6BC, *C. felis*, *C. caviae *and *C. abortus*. While use of an alternative start codon usually results in low expression in *E. coli*, its impact on efficiency of translation in *Chlamydia *is not known. Nevertheless like *E. coli*, most chlamydial genes rely on AUG as a start codon [16].

**Figure 1:**
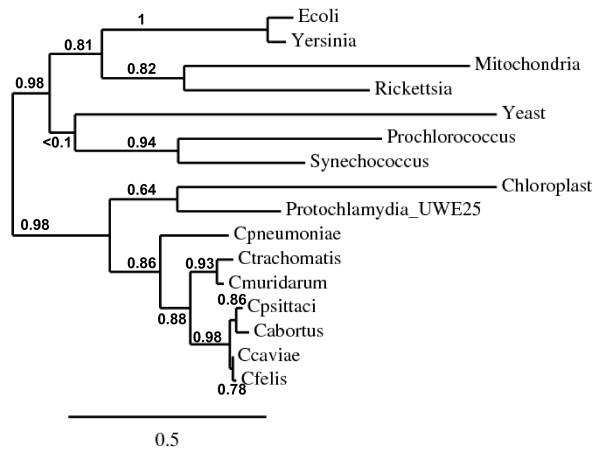
**Phylogenetic analysis of protein sequences showing the relationship between various KsgA orthologs**. Sequences were aligned and analyzed on the phylogeny.fr interface. Due to the conservation of KsgA sequences, only one representative of each chlamydial species was chosen for clarity of the tree. GenBank accession no.: NP_414593 (*Escherichia coli *K12), YP_069175 (*Yersinia pseudotuberculosis*), NP_057104 (transcription factor B1, mitochondrial [*Homo sapiens*]), CAA72482 (*Rickettsia prowazekii*), P41819 (Dim1p, *Saccharomyces cerevisiae*), YP_001017626 (*Prochlorococcus marinus *str. MIT 9303), YP_001734157 (*Synechococcus *sp. PCC 7002), NP_171690 (PFC-1, chloroplastic, *Arabidopsis thaliana*), YP_007394 (*Candidatus Protochlamydia amoebophila *UWE25), NP_445330 (*C. pneumoniae *AR39 ), YP_001654684 (*C. trachomatis *serovar L2), NP_297007 (*C. muridarum *Nigg ), GQ284731 (*C. psittaci *6BC), CAH63750 (*C. abortus *S26/3), NP_829174 (*C. caviae *GPIC ), YP_515617 (*C. felis *Fe/C-56)

A multiway alignment between various KsgA homologs revealed 62 to 68% identity amongst the *Chlamydiaceae *and at least 99% within each species, 39% with the protochlamydial homolog, 25 to 30% with *Arabidaposis thaliana*, *Synechococcus *sp., *Prochlorococcus *sp., *E. coli*, *Rickettsia *sp. and *Yersinia *sp. KsgA orthologs, and 20% with the mitochondrial transcriptional factor h-mtTFB1 (data not shown). Using a refined sequence alignment, a phylogenetic tree was constructed for these various dimethyltransferases [17] (see Materials and Methods). The branches representing *Chlamydia *were similar whether nucleotide sequences (data not shown) or protein sequences (Figure 1) were aligned. They were both comparable to the one obtained using 16S rRNA and 23S rRNA gene sequences [18] or housekeeping gene fragments [19], except that *C. pneumoniae *was slightly more distant in our analysis, with a strong branch support value of 86%.

**Figure 2:**
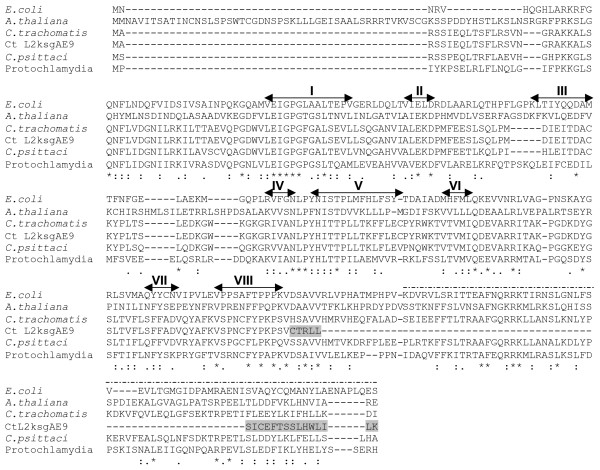
**Structure-based sequence alignment of the KsgA alleles from *E. coli *(NP_414593), *A. thaliana *(NP_171690 i.e. PFC1), *C. trachomatis *L2 (YP_001654684 i.e. CTL0608), *C. trachomatis *L2*ksgAE9*, *C. psittaci *6BC (GQ284731) and *Candidatus Protochlamydia amoebophila *UWE25 (YP_007394 i.e. pc0395)**. The alignment was generated using the Tcoffee expresso Web server using the structure of *E. coli *KsgA (PDB ID 1QRYR). Identical residues are denoted with an asterisk and strongly conserved residues with a colon; weakly conserved residues are marked with a period. Double-headed arrows indicate structural or catalytic motifs (I to VIII) common to S-adenosylmethionine-dependant methyltransferases and a dotted line indicates the C-terminal domain of *E. coli *KsgA [49]. Shading shows change in amino acid sequence in KsgA_L2ksgAE9 _resulting from frameship up to stop codon and truncation.

The chlamydial KsgA homologs, in particular the protochlamydial one, grouped with the *A. thaliania *chloroplastic PFC1 (Paleface1) protein, in agreement with two recent studies [4,20] (Figure 1). Although the evolutionary relationship between plants and *Chlamydia *may seem surprising considering that no species of *Chlamydia *has been reported in photosynthetic organisms, a high number of cyanobacteria- and plant-like genes in *Chlamydia *have been identified in the different chlamydial genome sequences [21,22]. Further analyses showed that many of the plant orthologs are targeted to the plastids in plants. We recently demonstrated that *C. trachomatis *L2 and *P. amoebophila *DapL enzymes behave similarly to the phylogenetically related plant plastidial LL-diaminopimelate aminotransferase enzyme involved in the lysine biosynthetic pathway [23]. Accordingly, we predict that the chlamydial KsgA proteins should be functionally equivalent to the plastid orthologs. In addition, conservation of hallmark sequences for binding to the methyl group donor S-adenosyl-L-methionine and to the adenine ring of the nucleotide to be methylated (Figure 2) predict that the chlamydial KsgA proteins should complement a KsgA defect in *E. coli*, as seen previously with KsgA family members [5,24-27].

### 2-Complementation of a *ksgA *mutant of *E. coli *by the chlamydial *ksgA *homologs

*E. coli *are naturally sensitive to KSM, an aminoglycoside antibiotic that inhibits the initial step of protein synthesis. Sensitivity to KSM is in part due to the action of KsgA which methylates the adenosine residues at positions 1518 and 1519 of the 16S rRNA. [Note that we use the *E. coli *numbering system throughout when referring to nucleotide(s) in rRNA genes]. Consequently, *ksgA *mutants are resistant to KSM [28]. We constructed a clean deletion of *ksgA *in *E. coli *MC4100 (Table 1) using the λ red recombinase method as described by Datensko and Wanner [29]. This strain, ATM809, was 16 times more resistant to KSM than the wild-type strain, with a minimal inhibitory concentration (MIC) of 2500 μg ml^-1 ^vs. 150 μg ml^-1 ^for its parent (Table 1). The ability of *E. coli *and *C. trachomatis *L2 *ksgA *genes to complement the KSM resistance phenotype and restore KSM sensitivity to ATM809 was first tested by transforming high copy number plasmids (Table 1) and expressing the respective genes in the presence of 1 mM Isopropyl β-D-1-thiogalactopyranoside (IPTG). Colonies formed by ATM809 transformed with pRAK316 (i.e. overexpressing *C. trachomatis *L2 KsgA protein, designated KsgA_L2 _for simplicity) were about 50% smaller than ATM809 or ATM809 transformed with pRAK297 (i.e. overexpressing the *E. coli *protein) (p < 0.0001 by t-test). The inhibition of growth due to overexpression of KsgA_L2 _made it impractical to assess sensitivity to KSM. To address this problem we constructed isogenic strains of ATM809 in which the copy number of the IPTG-inducible *ksgA *gene was reduced by inserting it into the chromosome at the λ-attachment site (Material and Methods). Under such conditions, we did not observe any growth inhibition associated with KsgA_L2 _expression in the absence of antibiotic (p > 0.1 by t-test).

**Table 1:** Bacterial strains used in this study and MICs for KSM

Strains	Description	Source or reference	KSM MIC (μg ml^-1^)
*E. coli*			
DH5α	F^-^ϕ80Δ(*lacZY-argF*)U169 *deoR recA1 endA1 phoA hsdR17 supE44 λ^- ^thi-1 gyrA96 relA1 Δ(lacZ)M15*	[56]	NT
BW25113	*Δ(araD-araB)567 ΔlacZ4787 (::rrnB-3) λ^- ^rph-1 Δ(rhaD-rhaB)568 hsdR514*	[29]	NT
MC4100	F- *araD139 *Δ(*argF-lac*)U169 *rpsL*150 *relA1 deoC1 rbsR fthD5301 fruA25 *λ^-^	[57]	150
ATM809	MC4100 Δ *ksgA::cat*	This work	2500
	ATM809 transformed with pRAK297 [*Plac::E. coli ksgA *in pGEMT]	This work	200
	ATM809 transformed with pRAK325 [*Plac:: C. psittaci 6BC UUG-ksgA *in pGEMT]	This work	1000
ATM810	ATM809 *att:: E.coli ksgA*	This work	200
ATM812	ATM809 *att:: C.trachomatis GUG-ksgA*	This work	800
ATM811	ATM809 *att:: C.trachomatis AUG-ksgA*	This work	400
ATM815	ATM809 *att:: C.trachomatis AUG-ksgAE9*	This work	2500

*C. psittaci *6BC	strain 6BC	T. Hatch	1100
BCK_1_	Spontaneous Ksm^R ^variant of *C. psittaci *6BC with A_794_G mutation in the 16S rRNA gene	[13]	>>5000

*C. trachomatis *L2	biovar lymphogranuloma venereum L2/434/Bu	H. Caldwell	800
L2 *ksgAE9*	*C. trachomatis *L2 *ksgAE9*	This work	3000

NT; Not Tested

**Figure 3:**
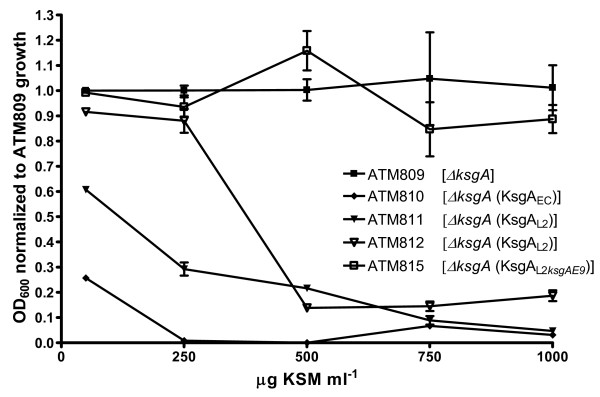
**KSM inhibition growth of *E. coli *ATM809 derivatives**. A *ksgA *mutant of *E. coli *(i.e. ATM809) harboring a chromosomal copy of *ksgA *from *E. coli *(ATM810), *C. trachomatis *L2 (ATM811, ATM812) or the KSM resistant *C. trachomatis *L2*ksgA*_E9 _(ATM815) were tested for resistance to KSM as described in [30], with some modifications. The A_600 _of cultures after 24 hrs of growth in the presence of the indicated amounts of KSM was normalized to the value obtained for ATM809 under the same conditions. Data points represent the mean ± s.d. of three experiments. The parent MC4100 behaved like ATM810 and was not included for clarity.

Expression of the native *E. coli *methylase in the *E. coli ksgA *mutant (i.e. ATM810) restored sensitivity to KSM as expected (Figure 3), with a MIC of 150 μg ml^-1 ^(Table 1). Similarly, expression of KsgA_L2 _was able to complement for the loss of *ksgA *in *E. coli *(Figure 3). As expected, the level of bacterial sensitivity to KSM was inversely related to the expected expression level of the recombinant protein in *E. coli*, lowering the MIC from 800 to 400 μg ml^-1 ^when the *ksgA_L2 _*GUG native start codon was replaced by the conventional AUG start codon to increase the level of expression of KsgA_L2 _(Table 1). Likewise expression of the *C. psittaci *6BC KsgA ortholog restored KSM sensitivity to the *E. coli ksgA *mutant (Table 1). The restoration of KSM sensitivity supports the conclusion that the chlamydial KsgA homologs are able to methylate the two conserved adenosines at positions 1518 and 1519 in *E. coli *16S rRNA *in vivo*, as has been seen previously for different KsgA family members from mitochondria, bacteria and yeasts [24,25,27,30,31].

**Figure 4:**
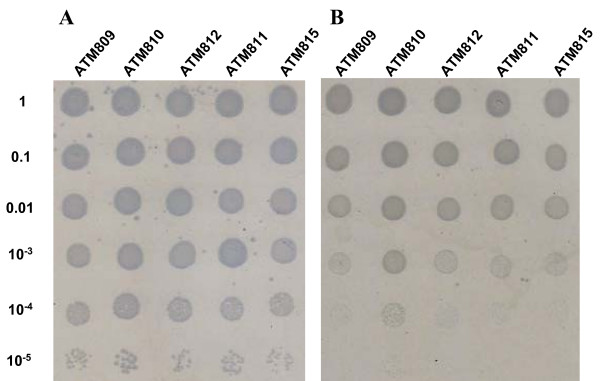
**Growth efficiency of various *E. coli *ATM809 derivatives at 30°C (panel A) and 20°C (panel B)**. Exponential cultures diluted in 10-fold increments were spotted on LB supplemented with IPTG and incubated at 30°C for 12 hrs and 20°C for 30 hrs.

The importance of KsgA in ribosome maturation has been linked to the cold-sensitivity phenotype described for *E. coli ksgA *mutants and *A. thaliana pfc1 *mutants [32,33]. Nevertheless expression of KsgA_L2 _in ATM809 did not reverse the cold-sensitivity growth of the *E. coli *strain (Figure 4). We do not know if this reflects a functional disparity of the chlamydial KsgA homolog or just results from partial heterologous complementation suggested by the intermediate level of KSM sensitivity exhibited by the *ksgA_L2_*-complemented *E. coli *mutants. Nevertheless, the ability of KsgA_L2 _to restore some degree of sensitivity towards KSM, hence methylation of A_1518 _and A_1519 _in *E. coli *16S rRNA, suggests that the same conserved adenosine residues are methylated in *Chlamydia *[3,6,33-36]. Consequently, we explored *Chlamydia *sensitivity to KSM.

### 3-Inhibition of *C. trachomatis *L2 and *C. psittaci *6BC growth by KSM and selection of resistant mutants

Although KSM has been used clinically in the treatment of *Pseudomonas aeruginosa *infections [37], this aminoglycoside antibiotic is only currently used in agricultural protection of rice crops against the fungus *Pyricularia oryzae*. Genetic, biochemical, and structural analyses have provided insights into KSM binding and inhibition [38-42]. The drug binds to the 30S ribosomal subunits within the path of the messenger RNA (mRNA), overlapping both the peptidyl-tRNA (P) and exit (E) sites. This affects the binding of the initiator tRNA onto canonical mRNAs and the subsequent joining of the large 50S subunits to form the translationally active 70S ribosomes. KSM is held in place by interactions with universally conserved residues A_794 _and G_926 _on the 16S rRNA and although the antibiotic does not appear to interact directly with the two adenosines at positions 1518 and 1519, lack of methylation in A_1519 _decreases KSM binding to rRNA. [39,40]. The conservation of these nucleotides in *Chlamydia *predicts that these obligate intracellular bacteria should be sensitive to KSM, provided that both the cell and the inclusion membrane are permeable to the drug.

In our laboratory, sensitivity of *Chlamydia *spp. to antibiotics is measured in the plaque assay and the MIC is defined as the concentration of drug that inhibits the development of 10^5 ^plaque-forming units (PFU). The number of input bacteria corresponds to a multiplicity of infection (MOI) of 0.01 in a confluent monolayer of L2 mouse fibroblasts in 60 mm dishes [10,11,13]. Due to low intracellular penetration, aminoglycosides are considered poorly active or not active against obligate intracellular bacteria and indeed *C. psittaci *6BC and *C. trachomatis *L2 MIC for KSM were high, i.e. 1100 and 800 μg ml^-1^, respectively (Table 1). Although such elevated MICs would classify the organisms as KSM resistant from a clinical stand point, we hypothesized that alterations in the drug binding site, in the KsgA methylase or in the rRNA methylation site would allow *Chlamydia *to grow in the presence of even higher concentrations of KSM, as reported for other bacteria [28,38,43,44].

When monolayers were infected with 10^6 ^to 10^7 ^PFU in the presence of KSM at 2000, 3000 or 5000 μg ml^-1^, resistant plaques appeared at a frequency of 2.20 ± 0.64 × 10^-5 ^for *C. psittaci *6BC while no plaques were seen for *C. trachomatis *under the same conditions (frequency <5.3 × 10^-8^). We have previously observed this difference in behavior between the two chlamydial strains for acquisition of resistance towards other ribosome targeting antibiotics [10,11]. Because expression of resistance requires that more than 50% of the ribosome population be of the resistant phenotype, *C. trachomatis *with two rRNA operons is at a disadvantage compared to *C. psittaci *6BC which harbors only one rRNA operon. KSM started to affect *C. trachomatis *L2 growth at 100 μg ml^-1^, but a concentration of 200 μg ml^-1 ^was completely inhibitory when cells were infected with a few hundred PFUs. Therefore we reasoned that serial passage in subinhibitory concentrations of antibiotic would allow the enrichment of putative low level KSM^R ^bacteria, as previously seen for azithromycin [10]. Three independent cultures of *C. trachomatis *L2 were passaged up to 14 times in KSM ranging from 100 to 800 μg ml^-1^. Three individual clones were then selected and purified in the plaque assay using 300 μg KSM ml^-1^.

### 4-Molecular and phenotypic characterizations of KSM resistance mutations in *C. psittaci *6BC and *C. trachomatis *L2

Sixteen independent spontaneous KSM^R ^plaques were isolated for *C. psittaci *6BC, expanded twice in the presence of 2000 μg KSM ml^-1 ^and further analyzed. DNA sequence analysis showed that none carried a mutation in *ksgA*. However, sequencing of the unique 16S rRNA gene revealed an A to G mutation at position 794 which is known as one of the binding sites for KSM [39,40]. Similarly, three *C. trachomatis *L2 isolates that arose in the presence of 300 μg KSM ml^-1 ^were further expanded in the presence of the drug and analyzed. None carried a mutation in the drug binding sites as expected from the presence of two rRNA operons in the strain [45]. None carried a mutation in *rpsI *encoding the 30S ribosomal subunit S9 protein that is a target for KSM resistance [46]. Sequencing of the *ksgA*_L2 _DNA region revealed the insertion of a *tg *nucleotide doublet in the last third of the gene. This doublet base-pair insertion, likely the result of a replication slippage [47], creates an early in-frame stop codon in the *ksgA *coding sequence, resulting in the expression of a KsgA variant of 213 residues instead of 277 (Figure 2).

Stability of the acquired phenotype was then tested on one representative mutant of both classes (Table 1) by growth in the absence of selective pressure (i.e. no KSM). Plaques formed by the *C. psittaci *6BC BCK_1 _and the *C. trachomatis *L2 *ksgAE9 *variants grown for a minimum of 14 days in the absence of selection displayed the same number of infectious particles when titered in the presence or absence of 2000 and 200 μg KSM ml^-1^, respectively, indicating that the resistance phenotype was stable (data not shown). Growth of *C. psittaci *BCK_1 _was not affected by 5 mg KSM ml^-1^, the highest concentration of KSM that was not toxic to the mouse fibroblast cells. On the other hand, plaques formed by *C. trachomatis ksgAE9 *were already smaller in the presence of 200 μg ml^-1 ^KSM, while 3 mg ml^-1 ^of the drug inhibited at least 10^5 ^*ksgAE9 *PFUs (i.e. MIC) (Table 1). The moderate level (i.e. less than 4-fold) of KSM resistance conferred by mutations in *ksgA *likely precluded *C. psittaci *6BC *ksgA *mutants from being selected directly in the plaque assay [10,11].

High level KSM resistance associated with mutations in the drug binding sites has only been observed previously in engineered strains of *E. coli *[38] and *B. subtillis *[44] in which each of the seven or ten rRNA operons, respectively, were inactivated, and the strains expressed only one rRNA operon encoded on a plasmid. Although both studies still reported that *ksgA *mutations are the main mechanism of resistance to KSM, mutations in the drug-binding site at position A_794 _and G_926 _of the 16S rRNA conferred four to eight times more resistance to KSM than KsgA inactivation or alteration of the adenosine targeted by the methylase at position 1519 of the 16S rRNA. In this study, all KSM^R ^mutations selected in *C. psittaci *6BC mapped to the drug-binding site at position A_794_G. Our inability to isolate KSM^R ^plaques for *C. psittaci *6BC with mutations in the 16S rRNA gene at position 926 or 1519 suggests that either they do not confer KSM resistance levels high enough to be selected in the plaque assay or they impose a significant burden on bacterial fitness. Such species-specific bias for drug resistance mutations has been reported for other ribosome-targeting antibiotics including tetracycline, linezolid and macrolides [48].

### 5-KsgA is critical for optimal growth of Chlamydia

Serial passages in sub-inhibitory concentrations of KSM allowed the purification of a *C. trachomatis *variant synthesizing a shortened rRNA adenine dimethyltransferase enzyme in which the eight structural and catalytic methyltransferase motifs were maintained, but harboring 20 unrelated amino acids because of the frameshift at codon 203 (Figure 2). Overexpression of the truncated chlamydial protein in ATM809, the *E. coli ksgA *mutant, was strongly inhibitory for cell growth, as observed earlier with the wild-type KsgA_L2 _protein. On the other hand expression from a copy of *ksgAE9*, the frameshift mutant, integrated in the bacterial chromosome, i.e. in ATM815, had no consequence on bacterial growth in the absence or in the presence of KSM (Figure 3), with a MIC of 2000 μg.ml^-1 ^(Table 1). This shows that the chlamydial KsgA C-terminal tail is essential for activity in this heterologous system. Crystal structure analyses revealed that the N-terminal and the C-terminal portions of *E. coli *and *Thermus thermophilus *KsgA orthologs form two domains and the presence of several positively charged residues in the C-terminal domain predicted its involvement in binding to rRNA [31,49]. Recent evidence has shown that both domains interact with 16S rRNA in 30S ribosomal subunits [42]. More specifically the C-terminal domain of KsgA competes with the C-terminal domain of initiation factor IF3 for binding with the central domain of 16S rRNA. Accordingly, KsgA would prevent ribosomes that are undergoing methylation from entering the translational cycle [42]. As a result one might expect immature ribosomes to enter translation in cells that lack KsgA thus bearing a physiological cost.

Screening for KSM resistance in *C. trachomatis *allowed us to select for *ksgA *mutations in these as yet genetically intractable pathogens [13]. Interestingly, plaques formed by the *C. trachomatis *L2 *ksgAE9 *variant in the absence of antibiotic were smaller than the wild-type strain (p < 0.0001 by t-test) with sizes of 0.27 mm ± 0.11 and 0.45 mm ± 0.13, respectively. This result shows that alteration in KsgA affects the fitness of *C. trachomatis in vitro*, similarly to *Mycobacterium tuberculosis *[50]. It is worth noting that although inactivation of *ksgA *has a minimal effect on growth of *E. coli *or *B. subtilis *at 37°C, recent studies have shown that the mutants are outcompeted by the wild-type strains at 37°C [44]. Similarly, while growth of a *Y. pseudotuberculosis ksgA *mutant is not severely affected under normal laboratory conditions, the mutant strain is attenuated in the mouse model indicating that dimethylation is essential for optimal fitness of the pathogen *in vivo *[51]. Although, the lack of a small animal model for *C. trachomatis *L2 precludes us from testing the consequences of KsgA inactivation on the pathogenicity of the strain, it is tempting to assume that the impairment of *C. trachomatis *L2 growth *in vitro *seen for our *ksgA *mutant would be reflected by an attenuation in virulence *in vivo*.

## Conclusions

When high throughput screening of compounds predicted to bind to *Bacillus subtillis *RNA methyltransferase ErmC identified new compounds that were inhibitory to the growth of *C. pneumoniae in vitro*, Alvesalo *et al. *proposed that these small compounds were targeting the bacterial KsgA homolog [52]. Considering that ribosome biogenesis factors are emerging as potential therapeutic targets to combat pathogens [32], the present work provides evidence that *Chlamydia *possess a functional rRNA dimethylase enzyme that is important for optimal growth of these obligate intracellular organisms. The demonstration of two methyltransferases in *Chlamydia *([53] and this study) raises the question of how these obligate intracellular bacteria obtain S-adenosyl-methionine to carry out these modifications. Our laboratory is currently investigating this aspect of *Chlamydia *biology.

## Methods

### Bacterial strains and antibiotics

The bacterial strains used in this study are listed in Table 1. *Escherichia coli *strain DH5α was used for cloning purposes. *E. coli *strains were grown in Luria-Bertani (LB) broth with aeration or on LB agar. Ampicillin (Sigma) was used at 100 μg ml^-1 ^to maintain plasmids in *E. coli *and at 25 μg ml^-1 ^to select for the ATM809 derivatives harboring a single copy of the pGEMT-insert integrated in the chromosome (Table 1). KSM (Biomol) was used at the concentrations indicated.

### Propagation of *Chlamydia *and tissue culture cells

*C. trachomatis *serovar L2/LGV/434/Bu and *C. psittaci *serovar 6BC were grown in mouse fibroblast L2 cells as previously described [11].

### Nucleic acid manipulation and sequence data analysis

Total genomic DNA was prepared from *C. trachomatis *L2 or *C. psittaci *6BC infected cells with DNeasy Tissue Kits (Qiagen). To identify the *ksgA *genomic region in *C. psittaci *6BC, a 5.6 kb product was amplified by PCR using degenerate primers designed from highly conserved genes surrounding the *ksgA *chromosomal region in the *Chlamydiaceae *(i.e. UpK-F1 [5'-CGACCACTCTGCCACTCTTCC-3'] and DWK-R1 [5'-CCYGTRATYTTWGCKATAGATCGTCGAGG-3']). The PCR product was cloned into pGEMT (Promega) and sequenced by the Biomedical Instrumentation Center at USUHS. Nucleotide sequences and predicted protein sequences were analyzed and aligned using Clone Manager 8 (Scientific & Educational Software, Durham, NC), focusing mainly on *ksgA *and its upstream gene, i.e. CT354 and CT355 respectively (*C. trachomatis *serovar D designation, GenBank accession number NC000117). Protein homolog searches used the bioinformatic tools provided by the National Center for Biotechnology Information (NCBI) and by the Berkeley Phylogenomics group http://phylogenomics.berkeley.edu/tools.php.

KsgA phylogenetic analysis was performed on the Phylogeny.fr platform [17] and comprised the following steps. Sequences were aligned with MUSCLE (v3.7) configured for highest accuracy (MUSCLE with default settings). After alignment, ambiguous regions (i.e. containing gaps and/or poorly aligned) were removed with Gblocks (v0.91b) using the following parameters: -minimum length of a block after gap cleaning: 10; -no gap positions were allowed in the final alignment; -all segments with contiguous non conserved positions bigger than 8 were rejected; -minimum number of sequences for a flank position: 85%. The phylogenetic tree was reconstructed using the maximum likelihood method implemented in the PhyML program (v3.0 aLRT). The WAG substitution model was selected assuming an estimated proportion of invariant sites (of 0.055) and 4 gamma-distributed rate categories to account for rate heterogeneity across sites. The gamma shape parameter was estimated directly from the data (gamma=1.612). Reliability for internal branch was assessed using the aLRT test (SH-Like). Graphical representation and edition of the phylogenetic tree were performed with TreeDyn (v198.3).

### Construction of the *E. coli *K12 ΔksgA mutant, ATM809

An *E. coli ksgA*-mutant was generated first in strain BW25113 (Table 1) using the λ Red recombinase method described by Datensko and Wanner [29]. The pKD4 template plasmid (chloramphenicol resistance) was used with the primers KD5 [5'-**CACCCAATGAATAATCGAGTCCACCAGGGCCACTTAGCCCGTAAACGC**tgtgtaggctggagctgcttc-3'] and KD6 [5'-**CGAATTGATCATCGTTAACTCTCCTGCAAAGGCGCGTTCTCCGCCA**catatgaatatcctccttagttcc-3']. The PCR product that contains 46 bp of DNA homologous to the *E. coli *chromosomal regions flanking *ksgA*, shown underlined in the above primers, was used to electroporate BW25113 containing the λ Red recombinase expression plasmid pKD46. Expression of the λ Red system allowed for recombination of the PCR product into the bacterial chromosome and replacement of the chromosomal *ksgA *copy by the chloramphenicol resistance gene. This mutation was confirmed using PCR analysis of genomic DNA using the primers ksgA-F3 [5'-ACCCAATGAATAATCGAGTCCACC-3'] and ksgA-R2 [5'-TGATCATCGTTAACTCTCCTGCAAAG-3']. Next, a P1L4 lysate was grown on the *ΔksgA::cat *strain and used to transduce MC4100 to chloramphenicol resistance, creating ATM809 (Table 1). Deletion of *ksgA *in ATM809 was confirmed by PCR analysis (see above) and resulted in increased resistance to KSM (Table 1).

### Cloning of *ksgA *in plasmid pGEMT

Complementation plasmids were constructed by cloning PCR products generated from *E. coli *or chlamydial DNA lysates into pGEMT (Promega), under the control of the lactose promoter. Sequencing of all cloned genes was performed by the Biomedical Instrumentation Center at USUHS. *E. coli *K12 *ksgA *gene was amplified using primers ksgA-F3 and ksgA-R2, creating pRAK297. *C. trachomatis *L2 *ksgA *gene was amplified using primers DWK-R2 [5'-ACTCAAGATCTCTAATCATAATCCCA-3'] and ksgA-F [5'-AGGGTGGCACGGAGTTCTATAGAAC-3'] containing the genuine GUG start codon or ksgAF5 [5'-GGATGGCACGGAGTTCTATAGAAC-3'] where the GUG start codon has been replaced by the conventional AUG start codon, creating pRAK317 and pRAK316, respectively. pRAK363 was constructed similarly to pRAK316 except that the PCR product was amplified from *C. trachomatis *L2 *ksgAE9 *genomic DNA (Table 1). *C. psittaci *6BC *ksgA *homolog was amplified using primers ksgA-F8 [5'-CGCTATGGCTTTGACTCATCG-3'] and ksgA-R6 [5'-CACTTAGGCGTGCAATGAGAG-3'] and cloned in pGEMT under the control of the lactose promoter, creating pRAK325. In this construct the putative KsgA_6BC _UUG start codon was ~ 60 bp downstream of the Ribosome Binding Site, and was consequently expected to be expressed at a low level in *E. coli *due to polar effect. Accordingly, unlike *E. coli *transformants harboring pRAK316, pRAK317 or pRAK363, we did not observe any growth defect in *E. coli *pRAK325 transformants after addition of IPTG (see below).

### Insertion of the *ksgA*-pGEMT inserts into ATM809 chromosome

We predicted that expressing KsgA_L2 _at a physiological level will reduce the growth inhibition observed in *E. coli *when the gene was carried on high copy vectors. Therefore inserts from plasmids pRAK297, pRAK316, pRAK317 and pRAK363 were inserted at the *att *site in the chromosome of ATM809 using the λlnCh tool as described by Boyd et al. [54]. Briefly, homology between the *ksgA*-pGEMT constructs (near-*ori *region and part of the *bla *gene) and sequences on the InCh bacteriophage allowed some phages to acquire both the ampicillin resistance gene (i.e. *bla*) and the vector *ksgA *insert by recombination. After subsequent infection of ATM809, the recombinant phage *bla *- *ksgA *region was transferred into the bacterial *att *site by site-specific recombination, creating lysogens. Finally recombination between the bacterial genome near *att *and the phage DNA that carry the same region resulted in deletion of the phage while leaving *bla-ksgA *in the bacterial genome, creating bacterial stable recombinants. Insertion sites were confirmed by PCR [54]. Primers Puc-F and Puc-R [10] showed the presence of the *ksgA *insert in ATM810, ATM811, ATM812 and ATM815 (Table 1). Primers GalF [5'-CTTGCTGAGTACGTGAGTTC-3'] and IG-R [5'-ACGTTGGAGTCCACGTTCTT-3'] amplified a 1248 bp product in ATM810, ATM811 and ATM815 only, showing that these three strains were stable recombinants. On the other hand, we were unable to obtain "stable" recombinants from ATM812, as seen by PCR amplification of a 977 bp product using primers GalF and Att-R [5'-AAGCAGGCTTCAACGGATTC-3'], similarily to MC4100 and ATM809. Maintenance of the *ksgA *insert in ATM812 chromosome was followed throughout the study by PCR with Puc-F and Puc-R and by the constant presence of ampicillin for selection.

### Analysis of bacterial growth

The effect of KsgA on bacterial growth was first studied in *E. coli *recombinants. Cells were grown at 37°C overnight in LB without salt [55] supplemented with ampicillin. Saturated cultures were diluted 1/100 in fresh expression medium (i.e. LB without salt supplemented with 1 mM IPTG) and incubated at 37°C for 2 to 3 hours, then normalized to an OD_600 nm _equivalent of 0.5. Bacteria were serially diluted in sterile buffered saline gelatin [150 mM NaCl, 2 mM KH_2_PO_4_, 4 mM Na_2_HPO_4_, 0.01% gelatin]. For each dilution, aliquots of 2 μl were spotted onto two expression medium agar plates. One plate was left at 20°C for about 30 hrs and the second plate was incubated at 30°C for 12 hrs (Figure 4). 100 μl of a 10^-5 ^dilution was plated on expression medium agar plates and incubated at 37°C for 12 hrs. The sizes of 15 random colonies were determined for each strain and averaged.

The effect of KsgA on *C. trachomatis *growth was determined by measuring and averaging the size of a minimum of 60 plaques formed by the two *C. trachomatis *L2 variants in the absence of selection, two weeks after simultaneous inoculation onto three 60-mm confluent monolayers of mouse fibroblast cells each.

### KSM sensitivity assays

MIC was determined for *E. coli *in test tubes inoculated with about 10^5 ^bacteria collected from exponential phase cultures grown at 37°C in expression medium containing various concentrations of KSM. MIC was defined as the lowest concentration at which no growth was visible after 16 hrs incubation at 37°C (Table 1). Subsequently, cultures were also incubated in triplicate with agitation for 24 hrs in the presence of 50, 250, 500, 750 and 1000 μg ml^-1 ^of KSM in expression medium. Growth was determined spectrophotometrically at 600 nm and normalized to the value obtained for ATM809 at the same concentration of KSM. KSM inhibition growth curves (Figure 3) were generated using the Prism 3.0 software (GraphPad Software, Inc, San Diego, CA).

Susceptibility of *C. trachomatis *and *C. psittaci *to KSM was examined in the plaque assay. MIC was defined as the drug concentration that inhibits the development of 10^5 ^chlamydial plaque-forming units (PFU) in a confluent L2 monolayer in a 60 mm dish [11]. To test for spontaneous drug resistance, 60 mm dishes were infected with 10^7 ^to 10^8 ^PFU (MOI 1 to 10) and the drug was added two hrs p.i. at a concentration high enough to inhibit the cytotoxicity associated with this inoculum size. The frequency of spontaneous mutation to drug resistance was determined by dividing the number of PFU on selective medium by the number of PFU added to the monolayer (as measured by titration of PFU in the absence of antibiotic) [11]. Three clonal *C. trachomatis *KSM^r ^isolates, including L2 *ksgAE9 *(Table 1) were independently purified in the plaque assay in the presence of 300 μg/ml of KSM, following 7 to 13 successive passages in mouse cells in the presence of increasing concentrations of antibiotic (from 100 to 800 μg ml^-1^).

### PCR and DNA sequencing of the KSM resistance targets in *Chlamydia*

PCR amplification and DNA sequencing were used to determine whether chlamydial resistance to KSM was due to a mutation in the 16S rRNA gene as described in [13] or in *ksgA *using the primers described above. Because *ksgA *is apparently the second gene of a bicistronic operon in *C. trachomatis *and *C. psittaci*, we also sequenced about 2 kb upsteam of *ksgA*. DNA sequences for each antibiotic resistant isolate were aligned using Clone Manager 8 and compared to the respective DNA sequence obtained from the wild-type parental strain. We also amplified *C. trachomatis *L2 *rpsI *region using primers rpsI-F1 [5'-GCTGAGAAAGTGCGTTTGACTG-3'] and rpsI-R1 [5'-GAAAGCAAGCACGGGACAAATC-3'] and sequenced the PCR fragment using primer rpsI-F2 [5'-ACATGATTGCGCGAAAGC-3']. No mutation was seen in the three KSM resistant isolates.

### Nucleotide sequence accession number

*C. psittaci *6BC *ksgA *sequence determined in the present study has been deposited in GenBank under accession number GQ284731.

## Authors' contributions

RB designed and conceived the study, conducted the experiments, analyzed results and wrote the manuscript. ATM analyzed results and revised the manuscript.

## References

[B1] Noller HF RNA structure: reading the ribosome Science 2005 309 1508 1514 10.1126/science.1111771 16141058

[B2] McCloskey JA Rozenski J The Small Subunit rRNA Modification Database Nucleic Acids Res 2005 33 D135 D138 10.1093/nar/gki015 15608163 PMC539969

[B3] Cunningham PR Weitzmann CJ Nurse K Masurel R Van Knippenberg PH Ofengand J Site-specific mutation of the conserved m62 A m62 A residues of *E. coli *16S ribosomal RNA. Effects on ribosome function and activity of the *ksgA *methyltransferase Biochim Biophys Acta 1990 1050 18 26 2207142 10.1016/0167-4781(90)90135-o

[B4] Park AK Kim H Jin HJ Comprehensive phylogenetic analysis of evolutionarily conserved rRNA adenine dimethyltransferase suggests diverse bacterial contributions to the nucleus-encoded plastid proteome Mol Phylogenet Evol 2009 50 282 289 10.1016/j.ympev.2008.10.020 19017544

[B5] McCulloch V Seidel-Rogol BL Shadel GS A human mitochondrial transcription factor is related to RNA adenine methyltransferases and binds S-adenosylmethionine Mol Cell Biol 2002 22 1116 1125 10.1128/MCB.22.4.1116-1125.2002 11809803 PMC134642

[B6] Lafontaine D Vandenhaute J Tollervey D The 18S rRNA dimethylase Dim1p is required for pre-ribosomal RNA processing in yeast Genes Dev 1995 9 2470 2481 10.1101/gad.9.20.2470 7590228

[B7] Corsaro D Venditti D Emerging chlamydial infections Crit Rev Microbiol 2004 30 75 106 10.1080/10408410490435106 15239381

[B8] Corsaro D Greub G Pathogenic potential of novel Chlamydiae and diagnostic approaches to infections due to these obligate intracellular bacteria Clin Microbiol Rev 2006 19 283 297 10.1128/CMR.19.2.283-297.2006 16614250 PMC1471994

[B9] Abdelrahman YM Belland RJ The chlamydial developmental cycle FEMS Microbiol Rev 2005 29 949 959 10.1016/j.femsre.2005.03.002 16043254

[B10] Binet R Maurelli AT Frequency of development and associated physiological cost of azithromycin resistance in *Chlamydia psittaci *6BC and *C. trachomatis *L2 Antimicrob Agents Chemother 2007 51 4267 4275 10.1128/AAC.00962-07 17908942 PMC2167982

[B11] Binet R Maurelli AT Frequency of spontaneous mutations that confer antibiotic resistance in *Chlamydia *spp Antimicrob Agents Chemother 2005 49 2865 2873 10.1128/AAC.49.7.2865-2873.2005 15980362 PMC1168699

[B12] Wernegreen JJ For better or worse: genomic consequences of intracellular mutualism and parasitism Curr Opin Genet Dev 2005 15 572 583 10.1016/j.gde.2005.09.013 16230003

[B13] Binet R Maurelli AT Transformation and isolation of allelic exchange mutants of *Chlamydia psittaci *using recombinant DNA introduced by electroporation Proc Natl Acad Sci USA 2009 106 292 297 10.1073/pnas.0806768106 19104068 PMC2629194

[B14] Griffiths E Ventresca MS Gupta RS BLAST screening of chlamydial genomes to identify signature proteins that are unique for the Chlamydiales, Chlamydiaceae, Chlamydophila and Chlamydia groups of species BMC Genomics 2006 7 14 10.1186/1471-2164-7-14 16436211 PMC1403754

[B15] Belland RJ Zhong G Crane DD Hogan D Sturdevant D Sharma J Beatty WL Caldwell HD Genomic transcriptional profiling of the developmental cycle of *Chlamydia trachomatis* Proc Natl Acad Sci USA 2003 100 8478 8483 10.1073/pnas.1331135100 12815105 PMC166254

[B16] Ma J Campbell A Karlin S Correlations between Shine-Dalgarno sequences and gene features such as predicted expression levels and operon structures J Bacteriol 2002 184 5733 5745 10.1128/JB.184.20.5733-5745.2002 12270832 PMC139613

[B17] Dereeper A Guignon V Blanc G Audic S Buffet S Chevenet F Dufayard JF Guindon S Lefort V Lescot M Claverie JM Gascuel O Phylogeny.fr: robust phylogenetic analysis for the non-specialist Nucleic Acids Res 2008 36 W465 W469 10.1093/nar/gkn180 18424797 PMC2447785

[B18] Everett KD Bush RM Andersen AA Emended description of the order *Chlamydiales*, proposal of *Parachlamydiaceae *fam. nov. and *Simkaniaceae *fam. nov., each containing one monotypic genus, revised taxonomy of the family *Chlamydiaceae*, including a new genus and five new species, and standards for the identification of organisms Int J Syst Bacteriol 1999 49 Pt 2 415 440 10319462 10.1099/00207713-49-2-415

[B19] Pannekoek Y Morelli G Kusecek B Morré SA Ossewaarde JM Langerak AA Ende A van der Multi locus sequence typing of Chlamydiales: clonal groupings within the obligate intracellular bacteria *Chlamydia trachomatis* BMC Microbiol 2008 8 42 10.1186/1471-2180-8-42 18307777 PMC2268939

[B20] Moustafa A Reyes-Prieto A Bhattacharya D Chlamydiae has contributed at least 55 genes to Plantae with predominantly plastid functions PLoS ONE 2008 3 e2205 10.1371/journal.pone.0002205 18493612 PMC2376095

[B21] Brinkman FS Blanchard JL Cherkasov A Av-Gay Y Brunham RC Fernandez RC Finlay BB Otto SP Ouellette BF Keeling PJ Rose AM Hancock RE Jones SJ Greberg H Evidence that plant-like genes in *Chlamydia *species reflect an ancestral relationship between Chlamydiaceae, cyanobacteria, and the chloroplast Genome Res 2002 12 1159 1167 10.1101/gr.341802 12176923 PMC186644

[B22] Horn M Collingro A Schmitz-Esser S Beier CL Purkhold U Fartmann B Brandt P Nyakatura GJ Droege M Frishman D Rattei T Mewes HW Wagner M Illuminating the evolutionary history of chlamydiae Science 2004 304 728 730 10.1126/science.1096330 15073324

[B23] McCoy AJ Adams NE Hudson AO Gilvarg C Leustek T Maurelli AT L,L-diaminopimelate aminotransferase, a trans-kingdom enzyme shared by *Chlamydia *and plants for synthesis of diaminopimelate/lysine Proc Natl Acad Sci USA 2006 103 17909 17914 10.1073/pnas.0608643103 17093042 PMC1693846

[B24] Cotney J Shadel GS Evidence for an early gene duplication event in the evolution of the mitochondrial transcription factor B family and maintenance of rRNA methyltransferase activity in human mtTFB1 and mtTFB2 J Mol Evol 2006 63 707 717 10.1007/s00239-006-0075-1 17031457

[B25] O'Farrell HC Pulicherla N Desai PM Rife JP Recognition of a complex substrate by the KsgA/Dim1 family of enzymes has been conserved throughout evolution RNA 2006 12 725 733 10.1261/rna.2310406 16540698 PMC1440906

[B26] Housen I Demonte D Lafontaine D Vandenhaute J Cloning and characterization of the KlDIM1 gene from *Kluyveromyces lactis *encoding the m2(6)A dimethylase of the 18S rRNA Yeast 1997 13 777 781 10.1002/(SICI)1097-0061(19970630)13:8<777::AID-YEA140>3.0.CO;2-1 9219342

[B27] Lafontaine D Delcour J Glasser AL Desgres J Vandenhaute J The DIM1 gene responsible for the conserved m6(2)Am6(2)A dimethylation in the 3'-terminal loop of 18 S rRNA is essential in yeast J Mol Biol 1994 241 492 497 10.1006/jmbi.1994.1525 8064863

[B28] Helser TL Davies JE Dahlberg JE Mechanism of kasugamycin resistance in *Escherichia coli* Nat New Biol 1972 235 6 9 4336392 10.1038/newbio235006a0

[B29] Datsenko KA Wanner BL One-step inactivation of chromosomal genes in *Escherichia coli *K-12 using PCR products Proc Natl Acad Sci USA 2000 97 6640 6645 10.1073/pnas.120163297 10829079 PMC18686

[B30] Seidel-Rogol BL McCulloch V Shadel GS Human mitochondrial transcription factor B1 methylates ribosomal RNA at a conserved stem-loop Nat Genet 2003 33 23 24 10.1038/ng1064 12496758

[B31] Demirci H Belardinelli R Seri E Gregory ST Gualerzi C Dahlberg AE Jogl G Structural rearrangements in the active site of the *Thermus thermophilus *16S rRNA methyltransferase KsgA in a binary complex with 5'-methylthioadenosine J Mol Biol 2009 388 271 282 10.1016/j.jmb.2009.02.066 19285505 PMC2679894

[B32] Connolly K Rife JP Culver G Mechanistic insight into the ribosome biogenesis functions of the ancient protein KsgA Mol Microbiol 2008 70 1062 1075 10.1111/j.1365-2958.2008.06485.x 18990185 PMC2709978

[B33] Tokuhisa JG Vijayan P Feldmann KA Browse JA Chloroplast development at low temperatures requires a homolog of DIM1, a yeast gene encoding the 18S rRNA dimethylase Plant Cell 1998 10 699 711 10.1105/tpc.10.5.699 9596631 PMC144018

[B34] Rife JP Moore PB The structure of a methylated tetraloop in 16S ribosomal RNA Structure 1998 6 747 756 10.1016/S0969-2126(98)00076-8 9655826

[B35] Guymon R Pomerantz SC Ison JN Crain PF McCloskey JA Post-transcriptional modifications in the small subunit ribosomal RNA from *Thermotoga maritima*, including presence of a novel modified cytidine RNA 2007 13 396 403 10.1261/rna.361607 17255199 PMC1800508

[B36] Guymon R Pomerantz SC Crain PF McCloskey JA Influence of phylogeny on posttranscriptional modification of rRNA in thermophilic prokaryotes: the complete modification map of 16S rRNA of *Thermus thermophilus* Biochemistry 2006 45 4888 4899 10.1021/bi052579p 16605256

[B37] Seiga K Iwata M Clinical application of Kasugamycin for *Pseudomonas aeruginosa *infections J Antibiot [B] 1967 20 184 186 4965582

[B38] Vila-Sanjurjo A Squires CL Dahlberg AE Isolation of kasugamycin resistant mutants in the 16 S ribosomal RNA of *Escherichia coli* J Mol Biol 1999 293 1 8 10.1006/jmbi.1999.3160 10512710

[B39] Schuwirth BS Day JM Hau CW Janssen GR Dahlberg AE Cate JH Vila-Sanjurjo A Structural analysis of kasugamycin inhibition of translation Nat Struct Mol Biol 2006 13 879 886 10.1038/nsmb1150 16998486 PMC2636691

[B40] Schluenzen F Takemoto C Wilson DN Kaminishi T Harms JM Hanawa-Suetsugu K Szaflarski W Kawazoe M Shirouzu M Nierhaus KH Yokoyama S Fucini P The antibiotic kasugamycin mimics mRNA nucleotides to destabilize tRNA binding and inhibit canonical translation initiation Nat Struct Mol Biol 2006 13 871 878 10.1038/nsmb1145 16998488

[B41] Woodcock J Moazed D Cannon M Davies J Noller HF Interaction of antibiotics with A- and P-site-specific bases in 16S ribosomal RNA EMBO J 1991 10 3099 3103 1915283 10.1002/j.1460-2075.1991.tb07863.x PMC453027

[B42] Xu Z O'Farrell HC Rife JP Culver GM A conserved rRNA methyltransferase regulates ribosome biogenesis Nat Struct Mol Biol 2008 15 534 536 10.1038/nsmb.1408 18391965

[B43] Duffin PM Seifert HS *ksgA *mutations confer resistance to kasugamycin in *Neisseria gonorrhoeae* Int J Antimicrob Agents 2008 33 4 321 7 10.1016/j.ijantimicag.2008.08.030 19097863 PMC2723803

[B44] Ochi K Kim JY Tanaka Y Wang G Masuda K Nanamiya H Okamoto S Tokuyama S Adachi Y Kawamura F Inactivation of KsgA, a 16S rRNA methyltransferase, causes vigorous emergence of mutants with high-level kasugamycin resistance Antimicrob Agents Chemother 2009 53 193 201 10.1128/AAC.00873-08 19001112 PMC2612157

[B45] Asai T Zaporojets D Squires C Squires CL An *Escherichia coli *strain with all chromosomal rRNA operons inactivated: complete exchange of rRNA genes between bacteria Proc Natl Acad Sci USA 1999 96 1971 1976 10.1073/pnas.96.5.1971 10051579 PMC26721

[B46] Dabbs ER *Escherichia coli *kasugamycin dependence arising from mutation at the *rpsI *locus J Bacteriol 1983 153 709 715 6337124 10.1128/jb.153.2.709-715.1983 PMC221688

[B47] Bichara M Wagner J Lambert IB Mechanisms of tandem repeat instability in bacteria Mutat Res 2006 598 144 163 16519906 10.1016/j.mrfmmm.2006.01.020

[B48] Binet R Maurelli AT Fitness cost due to mutations in the 16S rRNA associated with spectinomycin resistance in *Chlamydia psittaci *6BC Antimicrob Agents Chemother 2005 49 4455 4464 10.1128/AAC.49.11.4455-4464.2005 16251283 PMC1280162

[B49] O'Farrell HC Scarsdale JN Rife JP Crystal structure of KsgA, a universally conserved rRNA adenine dimethyltransferase in *Escherichia coli* J Mol Biol 2004 339 337 353 10.1016/j.jmb.2004.02.068 15136037

[B50] Tufariello JM Mi K Xu J Manabe YC Kesavan AK Drumm J Tanaka K Jacobs WR Jr Chan J Deletion of the *Mycobacterium tuberculosis *resuscitation-promoting factor Rv1009 gene results in delayed reactivation from chronic tuberculosis Infect Immun 2006 74 2985 2995 10.1128/IAI.74.5.2985-2995.2006 16622237 PMC1459759

[B51] Mecsas J Bilis I Falkow S Identification of attenuated *Yersinia pseudotuberculosis *strains and characterization of an orogastric infection in BALB/c mice on day 5 postinfection by signature-tagged mutagenesis Infect Immun 2001 69 2779 2787 10.1128/IAI.67.5.2779-2787.2001 11292689 PMC98225

[B52] Alvesalo JK Siiskonen A Vainio MJ Tammela PS Vuorela PM Similarity based virtual screening: a tool for targeted library design J Med Chem 2006 49 2353 2356 10.1021/jm051209w 16570931

[B53] Pannekoek Y Heurgue-Hamard V Langerak AA Speijer D Buckingham RH van der EA The N5-glutamine S-adenosyl-L-methionine-dependent methyltransferase PrmC/HemK in *Chlamydia trachomatis *methylates class 1 release factors J Bacteriol 2005 187 507 511 10.1128/JB.187.2.507-511.2005 15629922 PMC543528

[B54] Boyd D Weiss DS Chen JC Beckwith J Towards single-copy gene expression systems making gene cloning physiologically relevant: lambda InCh, a simple *Escherichia coli *plasmid-chromosome shuttle system J Bacteriol 2000 182 842 847 10.1128/JB.182.3.842-847.2000 10633125 PMC94354

[B55] Campbell BD Kadner RJ Relation of aerobiosis and ionic strength to the uptake of dihydrostreptomycin in *Escherichia coli* Biochim Biophys Acta 1980 593 1 10 10.1016/0005-2728(80)90002-X 6159001

[B56] Hanahan D Studies on transformation of *Escherichia coli *with plasmids J Mol Biol 1983 166 557 580 10.1016/S0022-2836(83)80284-8 6345791

[B57] Casadaban MJ Cohen SN Lactose genes fused to exogenous promoters in one step using a Mu-lac bacteriophage: *in vivo *probe for transcriptional control sequences Proc Natl Acad Sci USA 1979 76 4530 4533 10.1073/pnas.76.9.4530 159458 PMC411611

